# The Application of Genetic Risk Scores in Age-Related Macular Degeneration: A Review

**DOI:** 10.3390/jcm5030031

**Published:** 2016-03-04

**Authors:** Jessica N. Cooke Bailey, Joshua D. Hoffman, Rebecca J. Sardell, William K. Scott, Margaret A. Pericak-Vance, Jonathan L. Haines

**Affiliations:** 1 Department of Epidemiology and Biostatistics, Case Western Reserve University, Cleveland, OH 44106, USA; 2 Institute for Computational Biology, Case Western Reserve University, Cleveland, OH 44106, USA; 3 Department of Epidemiology and Biostatistics, University of California, San Francisco, CA 94158, USA; joshua.hoffman@ucsf.edu; 4 John P. Hussman Institute for Human Genomics, Miller School of Medicine, University of Miami, Miami, FL 33136, USA; r.sardell@med.miami.edu (R.J.S.); bscott@med.miami.edu (W.K.S.); mpericak@med.miami.edu (M.A.P.-V.)

**Keywords:** age-related macular degeneration (AMD), genetics, risk score

## Abstract

Age-related macular degeneration (AMD), a highly prevalent and impactful disease of aging, is inarguably influenced by complex interactions between genetic and environmental factors. Various risk scores have been tested that assess measurable genetic and environmental contributions to disease. We herein summarize and review the ability and utility of these numerous models for prediction of AMD and suggest additional risk factors to be incorporated into clinically useful predictive models of AMD.

## 1. Introduction

Age-related macular degeneration (AMD) is a significant contributor to global disease burden as one of the top three causes of irreversible blindness and visual impairment worldwide [1,2]. In the United States (US). AMD affects more than 11.5% of the population over the age of 80; this prevalence is higher among European Americans (>13.5% *vs.* ~2% for black, Hispanic, and “other” ethnic groups) [3]. Estimates indicate that nearly three million individuals in the US will develop AMD by the year 2020 [4] and, because AMD risk increases with age, AMD prevalence will continue to rise as the population of older individuals increases.

AMD is usually classified into early AMD (primarily excessive extracellular lipofuscinoid deposits (drusen) and advanced AMD. Advanced AMD is further divided into two forms: neovascular or “wet” AMD (choroidal neovascularization, (CNV)) which is distinguishable by formation and leakage of new blood vessels in the retinal pigment epithelial (RPE) layer of the macula; and non-neovascular “dry” AMD (also called geographic atrophy, (GA)), which is characterized by progressive RPE atrophy. Both forms of the disease can lead to irreversible vision loss. At present, clinical treatments, which exist only for CNV, fail to fully restore vision [5,6,7]. Additionally, despite the available treatment for CNV via intra-ocular injections of angiostatic agents that target vascular endothelial growth factor (VEGF), the key regulator of angiogenesis and vascular permeability in CNV progression (reviewed in [8]), fewer than 40% of patients have shown vision improvement with this treatment [5,6]. There is currently no effective therapy for non-neovascular AMD. Many other aspects of AMD also remain poorly understood; for instance, progression from early to late AMD varies between individuals but it is unclear what drives this variation [9,10]. With such a substantial disease burden and variable disease presentation, it is no surprise that AMD is one of several common diseases for which personalized and precision medicine are a priority; improved preventative strategies and detection methods for this disease are critical [8,11].

Genetic and epidemiological research has established the undeniable role of genetic variation in the etiology of AMD, with the heritable component estimated to be between 45% and 70% [12]. This is supported by twin studies reporting increased monozygotic *vs.* dizygotic twin concordance [12,13,14,15,16] and family studies reporting increased risk of 2–3 fold among first-degree relatives of patients [14,17,18]. Extensive genetic studies of AMD have identified several DNA variants that alter AMD risk. These studies include genome-wide linkage screens, candidate gene studies, and genome-wide association studies, (e.g., [19,20,21,22,23,24,25,26,27]). At this time, it is estimated that known genetic variants account for more than half of the heritability of AMD [28].

A genetic “risk score” (GRS) that calculates the cumulative genetic risk a person has for developing AMD based on their genotypes at variants known to be associated with risk of AMD could be a useful step toward the advancement of personalized and precision medicine. Additionally, several reports in the past decade have attempted to further unravel the complexity of AMD by examining interactions between factors that contribute to the development of AMD; whether these are clinically useful remains to be determined. Numerous reports have attempted to evaluate the use of AMD risk loci, sometimes in conjunction with environmental and demographic data, to assess AMD risk via analyzing case-control, cohort, and cross-sectional studies, which each have their own advantages and disadvantages with regards to assessing genetic risk (for review, please see [29,30]). This remains a controversial area, and it has been proposed that perhaps the age of personalized genomic profiling for AMD risk, among other diseases, has not yet been realized [31].

**Figure 1:**
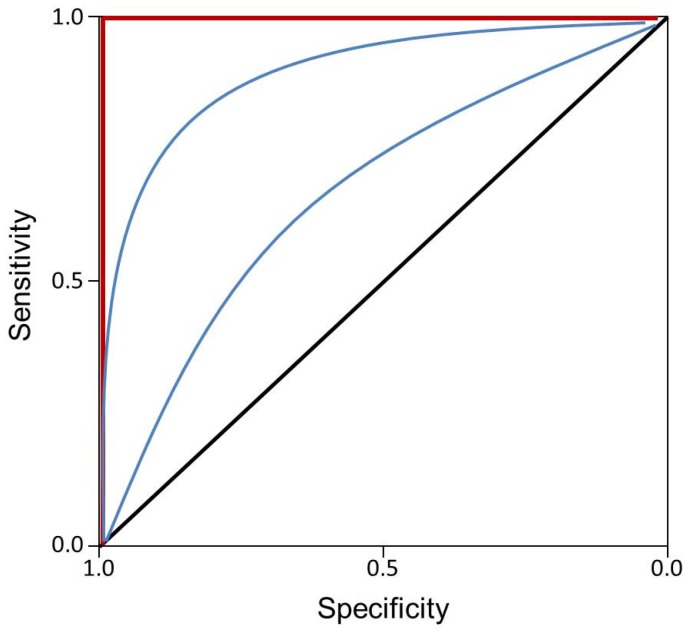
Example receiver operating characteristic (ROC) curve. Black line indicates area under ROC curve (AUC) = 0.5, or random chance. Red line indicates AUC = 1.0, or perfect model. Blue lines indicate varying ROC curves.

Risk scores have the potential to aid in evaluating the contribution of multiple factors to disease development and outcomes, progression, and response to treatment. Several risk models for AMD are based on genetics alone, whereas others include environmental, phenotypic, and/or demographic information. Results vary across studies and depend not only upon the population sample but the evaluated factors as well as the specific outcome of interest, *i.e.*, progression from early to late AMD, specific AMD subtype prediction, or general risk for AMD. Logistic regression models are utilized to assess factors contributing to risk of disease development in case-control studies and, in general, a standard way to evaluate these models is to assess the area under the receiver operating characteristic (ROC) curve (AUC), which is an indicator of model accuracy. The AUC statistic compares the true- (sensitivity) and false-positive (1-specificity) rates and is an indicator of the performance of predictive models, where an AUC = 0.5 indicates random chance, and AUC = 1 indicates a perfect model (Figure 1) [7]. The discriminatory power of the model to accurately categorize individuals determines whether it is a useful predictor—*i.e.*, whether the model will correctly predict the disease status of an individual, where sensitivity indicates the ability of the model to correctly predict individuals with the disease of interest and specificity indicates the ability of the model to accurately screen out individuals without the disease. Models are expected to have an AUC > 0.75 for informative screening of individuals who are at increased disease risk [7,32]. Obviously, the higher the AUC, the better the prediction and thus, the greater the clinical utility of the factors included in the model. An AUC of >0.7, for instance, is acceptable, while >0.9 is excellent. The disease or trait risk is highly dependent upon prevalence, which, for AMD, increases greatly with age, a factor that is often but not always included in predictive models [31]. Despite early reports that risk scores were not useful for prediction of AMD [31], a substantial number of AMD risk scores have been reported in the past five years with varying success, many with AUCs nearing 1; we herein summarize and review several of those models (Table 1).

## 2. Experimental Section

Various non-genetic factors contribute significantly to the development of AMD (reviewed in [44]). Riskscores that assess the utility of these factors in predicting disease development have therefore also been evaluated. Ying and colleagues evaluated non-genetic risk score models in the Complications of Age-Related Macular Degeneration Prevention Trial (CAPT) in participants with five years of follow up and with no GA at baseline [33]. The models estimated combinations of several baseline factors including age, smoking status, hypertension, night vision score, and Age-Related Eye Disease Study (AREDS) simple severity scale score to predict GA outcomes in patients with ≥10 large drusen and visual acuity ≥20/40 in each eye [33]. The best model included all five aforementioned factors and performed with an AUC of 0.76; this model evaluated the outcome of CAPT endpoint GA, defined as total area of GA (>250 u) in >1 disc area [33]. An updated non-genetic risk score was recently published by Chiu and colleagues that evaluated participants from the AREDS (mean 6.3 years follow-up) and Blue Mountains Eye Study (BMES; mean 10.5 years follow-up) without advanced AMD in at least one eye at baseline for progression to advanced AMD [34]. This model assessed the most consistent clinical risk factors: age, sex, education level, race, smoking status, pigment abnormality, soft drusen in the retina and maximum drusen size [34]. The model performed quite well with an AUC of 0.88 in the training set (AREDS), 0.91 in the validation set (BMES) [34].

With the heritability of AMD estimated between 45% and 70% [12,35], it is not unreasonable for AMD risk scores based solely upon participant genotype to have the potential to predict a substantial amount of disease risk. Various genetic-only AMD risk score models have been reported in recent years with overall success in discriminating individuals with AMD from unaffected individuals. Hageman and colleagues developed a genetics-only risk score for CNV using 13 AMD-associated SNPs in eight genes/loci (*CFH*, *CFHR4*, *CFHR5*, *F13B*, *C2*, *CFB*, *LOC287155*/*ARMS2*, *C3*) which they tested in a pooled group of 1132 cases and 822 controls from various studies. This model purposefully excluded self-reported and non-static variables to assess only the contribution of unchanging genetic factors to AMD and was tested and validated in independent subsets [41]. The AUC for the training set was 0.85, and for the validation set was 0.83; this model had 82% sensitivity and 63% specificity [41]. Interestingly, the group tested the 13-SNP model both with and without age and sex, but found no significant difference between the models [41]. Grassman and colleagues also approached the risk score from a genetics-only standpoint, assessing the ability of 13 SNPs at eight genetic loci (*CFH*, *ARMS2*, *CFB*, *C3*, *APOE*, *PLA2G12A*, *LIPC*, and *SYN3*/*TIMP3*) to predict AMD in a pooled sample of 986 (CNV, GA and CNV + GA) cases and 796 controls; this model yielded an AUC of 0.82 in the training set (2/3 of the total sample) and 0.81 in the validation set [42]. Further parsing the ability of the model to predict disease based on age group, sex, and AMD subtype yielded interesting results which indicated that younger AMD patients (<75 years) had significantly higher mean genetic risk score than older patients, suggesting that this risk score might have better performance in certain case groups [42]. Their findings support a model that classifies individuals based on five equally sized genetic risk score categories; disease status for individuals in the highest category is predictable with a sensitivity of 7.9%, but a specificity of 99.9% when compared to the four lower categories; only 2.2% of the individuals in the two lowest intervals are expected to develop AMD [42]. In the 2013 study of AMD by the AMD Gene Consortium, seven new loci were added to the list of 12 common variants associated with AMD and conducted a risk score analysis in a subset of the data with full SNP data at the 19 SNPs (7195 cases and 49,149 controls from 12 studies), evaluating the ability of these variants to predict AMD in each study that contributed data, using a leave-one-out cross-validation approach [35]. Weighting by the effect size of each allele, they evaluated the ability of the score to distinguish between AMD cases and controls [35]. The overall AUC for this model was 0.734, whereas for the seven novel SNPs alone it was 0.519 and for the 12 previously reported SNPs it was 0.736 [35]. Environmental variables were not included in the analysis, which would likely strengthen the power of the score. It is interesting that this model yielded a lower AUC than prior genetic-only models; given the large sample size of this study, this may more accurately reflect the predictive value of such a model. An updated study by this group, now the International AMD Genomics Consortium, reported 52 independent variants across 34 loci, adding 16 loci to the AMD risk locus list [28]. The updated risk score, which is also weighted proportional to the effect size of the variants, indicates that individuals in the highest decile of genetic risk, calculated have a 44-fold increased risk for advanced AMD compared to individuals in the lowest decile [28].

Because environment, demographic, lifestyle and genetic factors and their interactions influence AMD risk, it stands to reason that predictive models including many, if not all, of these factors are worth evaluating. Several groups have recently undertaken this research: Klein *et al.* developed a model for predicting advanced AMD that included age, smoking history, first-degree family history of AMD, modified AREDS phenotype at baseline, and major genetic variants *CFH* Y402H and *ARMS2* A69S in 2846 samples from the AREDS cohort with an average of 9.3 years of follow-up [36]. Their model performed well (AUC = 0.87 in initial sample) at predicting advanced AMD [36]. External validation was performed in the CAPT data, in which the Hosmer-Lemeshow statistic comparing the training and validation models was evaluated and was not statistically significant [36]. Similarly, Spencer and colleagues developed a model to predict individuals with high AMD risk based on age, smoking history, and genotypes at four separate loci (*CFH*, *CFB*, *ARMS2*, *C3*) in a training set; their best model performed similarly to other reported models with an AUC of 0.84 [32]. Seddon and colleagues presented various versions of similarly mixed risk models—the first incorporated age, gender, smoking history, BMI, education level, antioxidant treatment, genotype at six variants in five loci (*CFH*, *ARMS2*, *C2*, *CFB*, *C3*), AMD status at baseline, and drusen size in both eyes into a model to predict advanced AMD at a 5- and 10-year follow up in the AREDS study [37,45]; this model had an AUC of 0.90 in the test sample for progression to advanced AMD at 10 years. A follow-up model proposed by this group was also tested in a derivation sample comprised of AREDS samples and validated in a clinical population sample; this model differed from the earlier model in that antioxidant use was not included and the outcome, advanced AMD, was subsetted into GA and CNV to also evaluate the ability of the model to predict specific disease subtype [45]. The model had an AUC of 0.75 and 0.80 in the validation set for 5-year and 10-year progression, respectively. [45]. In their most recent risk score model, Seddon and colleagues included ten SNPs from loci independently associated with AMD progression (*CFH*, *ARMS2*/*HTRA1*, *CFB*, *C3*, *C2*, *COL8A1*, *RAD51B*, *C3*) in addition to age, sex, education, BMI, smoking, and baseline AMD status [38]. The genetic loci for this updated model include rare variant K155Q in the *C3* gene and evaluated 10-year progression to advanced AMD with an AUC of 0.90; the group also evaluated environmental and genetic factors separately and determined that the model incorporating both was significantly more accurate at predicting risk category [38].

Chen and colleagues also developed a predictive model of AMD using genetic and environmental factors; their best model included age, smoking status, and genotype from six AMD-associated variants in five genes (*CFH*, *CFB*, *HTRA1*/*LOC387715*, *C3*, *C2*) and had an AUC of 0.82 in their sample, which was not independently validated [39]. Though their risk model did not take into account difference in AMD type (GA or CNV), their SNP-level results suggest that this overall risk for development of specific disease subtype should be evaluated [39]. They suggest that risk scores for AMD should predict those individuals with early AMD at greatest risk for progression to advanced AMD (GA or CNV) and when that progression might occur [39]. In a more comprehensive model, Buitendijk *et al.* showed in a large sample of approximately 10,000 individuals with a mean follow-up of 11.1 years from the Three Continent AMD Consortium that a model including 26 SNPs in AMD risk genes, smoking, body mass index, and baseline AMD phenotype gave an AUC of 0.85 in the validation set [43]. This model outperformed their tested genetic-only and environment-only models by at least 6% (the second best model included genetic factors along with age and sex) [43].

GRS can also be used to examine the overall genetic architecture of a disease. For example, Hoffman *et al.* evaluated a genetic risk score generated using the 19 loci from Fritsche *et al.* [35] and determined that it was significantly lower in a sample of Amish individuals as compared to non-Amish European American AMD cases and controls, despite the similar prevalence of AMD in the groups [35,46]. This could indicate that additional genetic risk factors beyond those already identified exist in the Amish population. It also indicates that the currently established risk loci do not account for as large a proportion of AMD in this population [35,46], thereby justifying further studies in this group. Such studies will be useful for determining the additional genetic loci affecting disease prevalence and whether those variants have a detectable effect across larger and more diverse populations.

The GRS scores examined to date have been applied only to European Caucasian individuals. Additional studies in individuals of non-European descent are clearly necessary for assessing the utility of predictive models across ethnic groups, as the variants do not always directly translate, shown by studies that evaluated known AMD variants in African Americans and Mexican Americans and only detected association at the *ARMS2* A69S variant [47,48].

Moving the concept of GRS beyond prediction of AMD, several pharmacogenetic studies to assess the impact of genetics on various treatment and prevention strategies have been attempted. Awh and colleagues evaluated treatment response to antioxidants and zinc in the context of genotype at *CFH* and *ARMS2* loci using the AREDS study data; they assert that the genotype at these SNPs is important for predicting response to treatment [49,50]. Their study determined that patients progressed more with two risk alleles at the *CFH* locus and no risk alleles at the *ARMS2* locus when their AREDS treatment also contained zinc (compared with placebo) [49,50,51]. However, Chew and colleagues did not find any significant influence of genotype on relative treatment response to the AREDS supplement using the same dataset [52]. Smailhodzic and colleagues evaluated the cumulative effect of risk alleles at *CFH*, *ARMS2*, *VEGFA*, *KDR*, *LRP5*, and, *FXD4* in response to treatment with ranibizumab, the standard treatment for CNV [53]. This study evaluated the change in visual acuity between baseline and after three ranibizumab injections with respect to age of disease onset and genotype and found that high-risk alleles in *CFH*, *ARMS2*, and *VEGFA* were associated with younger age of onset of disease in combination with poor ranibizumab response [53]. In another study that incorporates treatment type, Perlee and colleagues evaluated 14 SNPs in *ARMS2*, *CFH*, *C3*, *C2*, *FB*, *CFHR4*, *CFHR5* and *F13B* along with BMI, AREDS treatment category (antioxidants only, antioxidants and zinc, zinc only, placebo), presence or absence of baseline advanced disease in nonstudy eye (phenotype), and education level for prediction of CNV and GA separately; their best model, with an AUC of 0.96 in the validation set, combined genotype and phenotype and performed equally as well as a model without demographic and environmental information in predicting CNV [40]. Important to note, however, is that baseline advanced AMD status in the non-study eye was incorporated into the model. Having advanced disease in one eye is therefore predictive of developing advanced disease in the other eye. These studies indicate that the incorporation of pharmacogenetic information into risk prediction relative to AMD may yet hold promise, though this remains controversial especially with regard to the AREDS supplements [49,50,51,52,54].

It is clear from the numerous AMD risk score models that one can be cautiously optimistic regarding the utility of such models in a clinical setting. The need for consistent reporting, use of training and validation sets for model building and testing, and realistic perspectives on the clinical implementation of these is apparent. Further, many reports of risk scores are not thorough in the factors explored; this is likely due to the lack of available information in the assessed samples. Future AMD risk scores should consider including demographic factors such as age, gender, and ethnicity, environmental/lifestyle risk factors such as smoking, BMI and cardiovascular risk factors (reviewed in [44]), AMD-associated biomarkers (e.g., [8,55,56]), and both risk and protective genetic variants (e.g., [57,58]), including common variants (e.g., [35]), rare variants such as those identified in *CFH*, *CFI*, *C3*, and *C9* [46,59,60,61,62,63], copy number variants [57,64,65,66], and mitochondrial variants [67]. In addition, the inclusion of relevant interaction terms could further increase the predictive ability of such models, evidenced by the significant interactions between genotype and smoking status reported by several groups (e.g., [68,69,70]). Genetic variants should also be weighted by the appropriate effect size to capture the true contribution of each variant, to elucidate an accurate representation of a patient’s true risk for AMD. As each of these entities has been postulated to modulate some fraction of risk for AMD, the clearest picture of whether risk scores are clinically useful would include testing all of the mentioned factors and evaluating models comparing them in order to assess what is essential to determining AMD risk in the most efficient but also realistic manner. Moreover, parsimoniously evaluating combinations that yield the highest sensitivity and specificity for prediction would be most useful. Various outcomes can be tested using these models including AMD overall, the likelihood as well as likely timeline to progression to late AMD, AMD subtype, eye concordance, and response to treatment. Developing a risk score for AMD that evaluates these components and contextualizes their clinical utility for predicting various scenarios—both prior to development of AMD and after early AMD is detected; as well as for treatment stratification—will make personalized and precision medicine more attainable. Studies proposing these models should discuss the predictive value of the tests including specific cutoff points at which each model is clinically useful for the outcome of interest.

## 3. Discussion

The glut of risk score literature is apparent. There are numerous factors that affect the performance of GRS and it is not likely that any single model gives a true idea of its predictive value, but rather an estimate in the subset of the general population that was studied. Importantly for AMD, the ages of patients, the specific genetic variants interrogated, the specific outcome evaluated, and the methods for evaluating demographic and lifestyle data vary by study; these factors can all impact the performance of predictive models, and therefore, comparing the models is not necessarily exacting. Phenotyping is the most timely part of any of the studies mentioned, and it has been shown that inter- and intra-individual variability exists in AMD grading [71]; consistency across and even within studies is certainly an issue that can affect the ability to detect risk signals and to devise valuable risk scores. Problematic for several of the studies reviewed here is that the same population sample (AREDS) was utilized [34,36,37,38,39,40,45,50,52,72] for model development, replication and/or validation. Additionally, implementing the maximal genetic-environment model may be difficult in situations where risk would ideally be assessed prior to becoming symptomatic for AMD. Because age is a major risk factor for the development of AMD and in fact defines this disease, assessment of patients (cases) and controls can be complicated by the potential that individuals may actually develop the disease later in life. The most accurately predictive models are those that include some form of baseline AMD indicator; however, in the absence of that information, for example in the case of a younger patient lacking symptoms of AMD, it is questionable whether a risk score that combines genetic and environmental/demographic information would be useful. It can be argued, however, that there would be clinical utility in a risk score that assesses one’s AMD progression speed/likelihood, one’s risk for developing GA or CNV, and one’s likelihood for benefitting from clinical intervention.

At the moment, genetic risk panels designed to clinically assess AMD risk do exist; the Macula Risk^®^ panel (Artic Medical Laboratory, Grand Rapids, MI, USA; [73]), for example, claims to “… identify, through genetic analysis, patients matching 35% of patients from the Age Related Eye Disease Study who had good outcomes with AREDS eye supplements but also another 13% of patients who experienced a detrimental effect due to the zinc component” [49,50]; unfortunately, as previously mentioned, the validity of the studies this panel is based on is in question [49,50,51,52,54]. Nicox markets the Sequenom^®^ RetnaGene AMD test (Sequenom Center for Molecular Medicine, Grand Rapids, MI, USA) to “evaluate the risk of a patient with early or intermediate AMD progressing to advanced choroidal neovascular disease within 2, 5, and 10 years ... based on five risk factors; genotype: the genetic profile of 12 disease-associated single nucleotide polymorphisms (SNPs), phenotype: baseline simplified severity scale grade 1, age, sex, and smoking status” as well as the RetnaGene AMD Lifetime Risk test (Sequenom Center for Molecular Medicine, Grand Rapids, MI, USA) to “evaluate the patient’s relative lifetime risk of developing advanced AMD, classified as lower than average, average, or higher than average risk … based on three risk factors; genotype: the genetic profile of 8 disease-associated single nucleotide polymorphisms (SNPs), age, and sex” [74]. Health insurance companies are aware of the existence of these panels and must determine the relevance to patients and the economic impact of providing coverage for these tests. The position statement of the Anthem^®^ BlueCross BlueShield company (San Francisco, CA, USA) is that these tests are “Investigational and Not Medically Necessary” [75].

## 4. Conclusions

The increasing prevalence and high morbidity associated with AMD and our increasing understanding of both genetic and non-genetic risk factors has encouraged exploration of risk modeling for AMD. Numerous models have been tested and most are significantly predictive of the measured outcome. However, there is substantial variability in the risk factors included, study designs used, and outcomes measured. For AMD risk scores to be truly valuable in the context of precision medicine, future studies must account for all of this variability and demonstrate utility across a wide spectrum of patients.

## Figures and Tables

**Table 1:** Review of genetic risk scores to predict age-related macular degeneration.

Genetic Loci	Environmental/Demographic/Lifestyle Data	Outcome	Predictive Ability in Initial Sample	Predictive Ability in Validation Sample	Study
None	Age, smoking status, hypertension, AREDS simple severity score, night vision score	Geographic atrophy	0.76	x	[33]
None	Age, sex, education level, race, smoking status, pigment abnormality presence, soft drusen, maximum drusen size	Advanced AMD	0.88	0.91	[34]
*CFH*, *ARMS2-HTRA1*, *C2-CFB*, *C3*	None	AMD	0.734		[35]
*CFH*, *ARMS2*/*HTRA1*	Age, smoking hx, first degree family hx of AMD, modified AREDS phenotype	Advanced AMD	0.872	Hosmer-Lemeshow statistic = 15.00, *p* = 0.09	[36]
*CFH*, *ARMS2*/*HTRA1*, *CFB*, *C3*	Age, smoking history	AMD high risk	0.84	x	[32]
*CFH*, *ARMS2*/*HTRA1*, *C2*, *CFB*, *C3*	Age, smoking, BMI, antioxidant treatment, presence of advanced AMD in one eye, drusen size in both eyes	10-year progression to advanced AMD	0.915	0.908	[37]
*CFH*, *ARMS2/HTRA1*, *C2*, *CFB*, *C3*, *COL8A, RAD51B*	Smoking status, BMI (adjusted for age, sex, education, 4 AREDS treatment groups)	10-year progression to advanced AMD	0.911	0.907	[38]
*CFH*, *C2*, *CFB*, *HTRA1*/*LOC387715*, *C3*	Age, smoking status	AMD	0.82	x	[39]
*CFH*, *CFHR4*, *CFHR5*, *F13B*, *C2*, *CFB*, *ARMS2*, *C3*, *HTRA1*	Baseline grade (AREDS simplified severity scale), smoking, BMI, age, sex, treatment (AREDS vitamin-mineral treatment), education	CNV/GA	CNV 0.96; GA 0.94		[40]
*CFH*, *CFHR4*, *CFHR5*, *F13B*, *C2*, *CFB*, *LOC387155*/*ARMS2*, *C3*	None	CNV	0.82	0.80	[41]
*CFH*, *ARMS2*, *CFB*, *C3*, *APOE,* *PLA2G12A*, *LIPC*, *SYN3*/*TIMP3*	None	AMD	0.820	0.813	[42]
*CFH*, *ARMS2*, *C2*/*CFB*, *C3*, *CFI*, *LPL*, *LIPC*, *MYRIP*, *SKIV2L*, *ABAC1*, *CETP*, *TIMP3*, *VEGFA*, *COL8A1*, *TNFRSF10A*, *FRK*/*COL10A1*, *SLC16A8*, *ADAMTS9*, *TGFBR1*, *RAD51B*, *IER3/DDR1*, *B3GALTL*	Age, sex, smoking, BMI, baseline AMD phenotype	AMD	0.88	0.85	[43]

AMD: age-related macular degeneration; CNV: choroidal neovascularization; GA: geographic atrophy; AREDS: age-related eye disease study; BMI: body mass index.
